# On Nervous or Convulsive Cough

**Published:** 1850-02

**Authors:** 


### PATHOLOGY AND PRACTICE OF MEDICINE.

*On Nervous or Convulsive Cough.* By M. SANDRAS.—There are several varieties of this:—1. The patient can receive no physical or moral impression, without suffering from a cough almost convulsive in its character. In examining the chest of such a person, the physician may be led into grievous error, and the unnecessary fear of incipient phthisis, unless he examines it on various occasions and under different circumstances. Patients with incipient phthisis also cough from the slightest cause; but it will be generally found that in those cases the impression is physical, while in those we are alluding to it is oftener moral.

2. Another form of cough, having some analogy to this, is observed whenever certain functions are brought into play, or when they are more actively exerted than usual. Thus, it is found in some whose meals have been too long delayed, in others as soon as they have eaten, especially if rather fully. Other persons cannot take a little extra muscular exertion without bringing on a tormenting cough of this kind. In both this and the preceding form the cough is dry and capricious, exhibiting very inconstant physical signs; but this latter form is somewhat more fixed in character than the first, inasmuch as in the same person it is always when the same function is fulfilled that it is produced; and it seems, too, to be more dependent upon disorder of the organs in connexion with the exercise of whose functions it appears; and this should be our chief guide for its treatment.

3. Another cough is observed upon the slightest irritation of the bronchi being produced; so that the least cold brings on a convulsive cough nearly as bad as that of pertussis. Sometimes, and especially in children and in very young adults, it takes on this form from the very commencement of the cold, and retains it until coction is produced. Each paroxysm is accompanied by a dry, raucous sound, and attempts at vomiting. Sometimes it is periodical, the disease only gradually assuming the characters of an ordinary ripening catarrh. In other cases the spasmodic character is only observed as the cough is drawing towards an end. Instead, however, of coction taking place, the expectoration continues frothy and transparent, and is only ejected by convulsive efforts and vomiting—the paroxysm being brought on by the slightest cause, and a state of spasmodic suffocation being almost induced, until a little transparent and frothy matter is expectorated, when all becomes quiet and normal until a new paroxysm. In some cases the cough suddenly ceases, without the expectoration having undergone any change; but this is rare. The causes of this pertussoid cough are not of easy appreciation. At the commencement, all is like a common cold; and it is the reiterated catching cold in an eminently neuropathic subject that seems to induce the aggravation. The prognosis, as regards immediate danger, is favorable; but is more serious in respect to future consequences, owing to the various evil consequences which may ensue upon the congestions the paroxysms give rise to. The destruction of sleep and disturbance of digestion which it causes are other important circumstances. Among the more serious results, is the production of hernias and of emphysema pulmonum. The irritation of the glottis and larynx should be relieved by tepid aqueous or narcotic vapours, and by the use of demulcent emulsions with laurel-water. When the expectoration is difficult, syrup of poppies, with small doses of tartar emetic, should be given, the antimony, whether it causes vomiting or not, affording great relief. So, too, small doses of extract of belladonna every night, or night and morning, should be given when the expectoration is somewhat modified, and in a few days the convulsive character of the cough usually abates. When this drug disagrees with the patient, it should be used endermically.

4. This variety may be called *hysterical*, from its occurring in hysterical patients. In a subject whose respiratory organs are habitually in a good condition, all at once an irregularly paroxysmal cough comes on, occurring at frequent intervals, and sometimes almost without intermission. It does not terminate with the expulsion of mucosities, but is either dry and objectless, or is accompanied by a true phlegmorrhagia. Hysterical phenomena sometimes precede or accompany the cough; while at others it ceases instantly that these appear. The cough is found to get worse and worse, in proportion to the developement of the hysteria; and this without any physical explanation of its intensity. The pulse is not febrile, but may be irregular, and such a one as is found in nervous subjects. The prognosis is favorable, unless the cough is mistaken for a phlegmasia, and aggravated by maltreatment. The treatment is, in fact, that which is proper for hysteria; but two means are especially indicated—the use of belladonna, and the employment of baths. Belladonma, given in doses of one seventh of a grain every half-hour, is highly efficacious; and it is rare for five or six doses to be given before improvement is visible. Baths at from 84° to 89° act as if by enchantment; but sometimes it is useful to give them at 75° to 82°; and this is the temperature which will in most cases prove the best, after the patient has already employed the higher.—*British and Foreign Med. Rev. from Bulletin de Thérapeutique.*

